# Supplementary material for: The Impact of Melatonin Supplementation and NLRP3 Inflammasome Deletion on Age-Accompanied Cardiac Damage
Source: Antioxidants (Basel). 2021 Aug 10;10(8):1269. doi: 10.3390/antiox10081269 (PMC8389221; doi:10.3390/antiox10081269)
Supplement: Supplementary file 1 [file antioxidants-10-01269-s001.zip › antioxidants-1313710-supplementary.pdf]

antioxidants

Supplementary Materials: The Impact of Melatonin Supplementation and NLRP3 Inflammasome Deletion on Age-Accompanied Cardiac Damage

Ramy K. A. Sayed [1,2,†], Marisol Fernández-Ortiz [1,†], Ibtissem Rahim [3], José Fernández-Martínez [1], Paula Aranda-Martínez [1], Iryna Rusanova [1,4], Laura Martínez-Ruiz [1], Reem M. Alsaadawy [5], Germaine Escames [1,4] and Darío Acuña-Castroviejo [1,4,6,*]

[1] Centro de Investigación Biomédica, Departamento de Fisiología, Facultad de Medicina, Instituto de Biotecnología, Parque Tecnológico de Ciencias de la Salud, Universidad de Granada, 18016 Granada, Spain; ramy.kamal@vet.sohag.edu.eg (R.K.A.S.); sol92@correo.ugr.es (M.F.-O.); josefermar@ugr.es (J.F.-M.); ampaula@correo.ugr.es (P.A.-M.); irusanova@ugr.es (I.R.); lauramartinezruiz8@gmail.com (L.M.-R.); gescames@ugr.es (G.E.)
[2] Department of Anatomy and Embryology, Faculty of Veterinary Medicine, Sohag University, Sohag 82524, Egypt
[3] Département de Biologie et Physiologie Cellulaire, Faculté des Sciences de la Nature et de la Vie, Université Blida 1, Blida 09000, Algeria; rahim.im15@gmail.com
[4] CIBERfes, Ibs.Granada, 18016 Granada, Spain
[5] Department of Zoonoses, Faculty of Veterinary Medicine, Assiut University, Assiut 71526, Egypt; reem.barbary@vet.au.edu.eg
[6] UGC de Laboratorios Clínicos, Hospital Universitario San Cecilio, 18016 Granada, Spain
* Correspondence: dacuna@ugr.es; Tel.: +34-95-824-1000 (ext. 20169)
† Authors contributed equally.

Table S1. List of primers used in RT-PCR assay.

Gene Symbol	Gene Description	Forward Primer	Reverse Primer
B-MHC	Myosin, heavy polypeptide 7, cardiac muscle, beta	CAAGCGGAAGCTGGAGGGA	CCTCGATGCGTGCCTGAAG
IL-1α	Interleukin 1 alpha	AGCCCGTGTTGCTGAAGGAGT	CCGACTTTGTTCTTTGGTGGCA
IL-6	Interleukin 6	AAAGCCAGAGTCCTTCAGAGAGA	GGAGAGCATTGGAAATTGGGGTA
TNF-α	Tumor necrosis factor alpha	AGCCCACGTCGTAGCAAACC	GGTGAGGAGCACGTAGTCGG
β-actin	Beta-actin	GCTGTCCCTGTATGCCTCTG	CGCTCGTTGCCAATAGTGATG
